# Reviewer acknowledgement 2013

**DOI:** 10.1186/2050-6511-15-33

**Published:** 2014-07-21

**Authors:** Helena C Flemyng

**Affiliations:** 1 BioMed Central Ltd., (236 Gray's Inn Road), London (WC1X 8HB), UK

## Abstract

**Contributing reviewers:**

The editors of BMC Pharmacology and Toxicology would like to thank all our reviewers who have contributed to the journal in Volume 14 (2013).

## 

Farzaneh Aghahosseini

Iran

Shahin Akhondzadeh

Iran

Catrin Albrecht

Germany

Samir Aleryani

United States of America

Salvatore Amoroso

Italy

Yuichi Ando

Japan

Sabrina Angelini

Italy

Eun Ju Bae

South Korea

Robert Bähring

Germany

Douglas Ball

Philippines

Desmond Bannon

United States of America

Dipika Bansal

India

Valerie Bardet

France

Jagadeesh Bayry

France

Rebecca Benham

United States of America

Carlos Bertoncini

Spain

Denise Boudreau

United States of America

David Boulton

United States of America

Jorgen Bramness

Norway

Thomas Brunner

Germany

Peter Buchwald

United States of America

Rene C Gaudreault

Canada

Paolo Calabresi

Italy

Davide Calebiro

Germany

Carlo Maria Ferdinando Caravaggi

Italy

Pablo Carbonell

France

A Brent Carter

United States of America

Michael Carvan

United States of America

Sarah Cavanaugh

United States of America

Alice Ceacareanu

United States of America

Laura Cerchia

Italy

Jeremiah Chakaya

Kenya

Thomas Chan

Hong Kong

Jeremy Chancellor

United Kingdom

Yoon-Seok Chang

South Korea

Keping Chen

China

Xingguo Cheng

United States of America

Gerardo Chowell

United States of America

Antonio Clavenna

Italy

Francesco Clementi

Italy

Joshua Courter

United States of America

Jordan Crago

United States of America

Cesare Cremon

Italy

Rosalia Crupi

Italy

Domenico Cucinotta

Italy

Jan De Waele

Belgium

Eric Deconinck

Belgium

Antonello Di Paolo

Italy

Eric Dietrich

United States of America

Tatiana Dilla

Spain

Dongyi Du

United States of America

Vangelis Economou

Greece

Patrick Erah

Nigeria

David Farb

United States of America

Hui Feng

United States of America

Juan Fernandez-Recio

Spain

Mark Fineman

United States of America

Philip Fowler

United Kingdom

Charles Frost

United States of America

Valentina Galbiati

Italy

Peter Galettis

Australia

Antonio Garcia

Spain

Pedro Gascon

France

Maria Cristina Gaudiano

Italy

Edward Goldstein

United States of America

Santhi Gorantla

United States of America

Rafael Gorodischer

Israel

Andrew Lofts Gray

South Africa

Matthew Grissinger

United States of America

Tyler Gums

United States of America

Abbas Haghparast

Iran

Shuntaro Hara

Japan

Kenji Hashimoto

Japan

Dan Hawcutt

United Kingdom

Lisa Hines

United States of America

Howard Hochster

United States of America

Sebastian Hoffmann

Germany

Hans Hogerzeil

Switzerland

Jonathan Hoggatt

United States of America

Kathleen Holloway

Switzerland

Yan Huang

United States of America

Tsuneya Ikezu

United States of America

Kiyotoshi Inenaga

Japan

Arantxa Isla

Spain

Takahiro Iwamoto

Japan

Evelyne Jacqz-Aigrain

France

Aminah Jatoi

United States of America

Paul Jurasz

Canada

Theodoros Kelesidis

United States of America

Yongpil Kim

Japan

Gil Klinger

Israel

Michael Kostapanos

Greece

Peter Kruger

Australia

Chun Shing Kwok

United Kingdom

Cornelia Landersdorfer

Australia

Vincenzo Lariccia

Italy

Marzia Lazzerini

Italy

Hans Lennernas

Sweden

Jason Li

United States of America

Claude Libert

Belgium

Kenneth Litwak

United States of America

Evelyn Lobo

United States of America

Jeffrey Lombardo

United States of America

Henrik Lövborg

Sweden

Hong Lu

United States of America

Kabirullah Lutfy

United States of America

Simona Magi

Italy

Rangan Maitra

United States of America

Madeeha Malik

Pakistan

Tadeusz Malinski

United States of America

Maura Marcucci

Canada

Raphael Margueron

France

Ataulfo Martinez-Torres

Mexico

Jason Matthews

Canada

Thomas Mavromoustakos

Greece

Patrick McDonnell

United States of America

Edy Meiyanto

Indonesia

Songshu Meng

China

Chieko Mineo

United States of America

Joseph Moellman

United States of America

Afzal Mohammed

United Kingdom

Kathryn Momary

United States of America

Bertrand Morel

Spain

Laura Moro

Italy

Gianluca Moroncini

Italy

Akira Murakami

Japan

Peter Nanasi

Hungary

Ajit Narang

United States of America

Eiji Nemoto

Japan

Detlef Neumann

Germany

Paul Newton

Laos

Charles Nhachi

United Kingdom

Daniel O’Connor

Australia

Michael Oldham

United States of America

Abdullah Olgun

Turkey

Chin Eng Ong

Malaysia

Bello Oricha

Nigeria

Cem Ozgonul

Turkey

Paulo Paixão

Portugal

Narayanan Parameswaran

United States of America

Ashish Pathak

India

Biniam Paulos

Ethiopia

Robert Pechick

United States of America

Claudia Pellacani

Italy

Giancarlo Pepeu

Italy

Gary Perdew

United States of America

Bertram Pitt

United States of America

Vincent Poitout

Canada

Aristides Polyzos

Greece

Brenda Porter

United States of America

Alessandra Pugi

Italy

Thiyagu Rajakannan

United States of America

Marcia Ratner

United States of America

Marcus Reidenberg

United States of America

Carlo Riccardi

Italy

Jason Roberts

Australia

John Roberts

United States of America

Jane Robertson

Australia

Arlin Rogers

United States of America

Simona Ronchetti

Italy

Bernd Rosenkranz

Suriname

Emilio Russo

Italy

Tiina Saarto

Finland

Qayyim Said

United States of America

Luigi Santambrogio

Italy

Sam Schulman

Canada

Dietmar Schwab

Switzerland

Yutaka Seino

Japan

Sang Mi Shin

South Korea

Si Mui Sim

Malaysia

Nicolas Simon

France

Anurag Singh

United States of America

Daniel Smart

Switzerland

Dave Smith

Sweden

Olivier Sperandio

France

Karl Staples

United Kingdom

Douglas Steinke

United Kingdom

Chris Stockmann

United States of America

Nithima Sumpradit

Thailand

Olivier Taboureau

France

Stanley Tahara

United States of America

Giovanni Tarantino

Italy

Maria Thurston

United States of America

Mondher Toumi

France

Marie Tournier

France

Luigi Uccioli

Italy

Mikko Uusi-Oukari

Finland

Eric Vanderploeg

United States of America

Rick Vega

United States of America

Elisabetta Verderio Edwards

United Kingdom

Andreas Vilhelmsson

Sweden

Chris Vulpe

United States of America

Cheryl Watson

United States of America

Björn Wettermark

Sweden

Zhigang Xie

United States of America

Fadi Xu

United States of America

Jialin Xu

United States of America

Hsin-Sheng Yang

United States of America

Zhiyong Yang

United States of America

Tzung-Hai Yen

Taiwan

Ulrich Zachariae

United Kingdom

Ming Zhan

United States of America

Jianli Zhang

China

Liming Zhou

China

Frans Zitman

Netherlands

Claudia Zwingmann

Canada

## Pre-publication history

The pre-publication history for this paper can be accessed here:

http://www.biomedcentral.com/2050-6511/15/33/prepub

